# Presenting simulation results in a nested loop plot

**DOI:** 10.1186/1471-2288-14-129

**Published:** 2014-12-12

**Authors:** Gerta Rücker, Guido Schwarzer

**Affiliations:** Institute for Medical Biometry and Statistics, Medical Center – University of Freiburg, Stefan-Meier-Strasse 26, 79104 Freiburg, Germany

**Keywords:** Simulation study, Plot, Diagram, Graphical representation, Trellis plot

## Abstract

**Background:**

Statisticians investigate new methods in simulations to evaluate their properties for future real data applications. Results are often presented in a number of figures, e.g., Trellis plots. We had conducted a simulation study on six statistical methods for estimating the treatment effect in binary outcome meta-analyses, where selection bias (e.g., publication bias) was suspected because of apparent funnel plot asymmetry. We varied five simulation parameters: true treatment effect, extent of selection, event proportion in control group, heterogeneity parameter, and number of studies in meta-analysis. In combination, this yielded a total number of 768 scenarios. To present all results using Trellis plots, 12 figures were needed.

**Methods:**

Choosing bias as criterion of interest, we present a ‘nested loop plot’, a diagram type that aims to have all simulation results in one plot. The idea was to bring all scenarios into a lexicographical order and arrange them consecutively on the horizontal axis of a plot, whereas the treatment effect estimate is presented on the vertical axis.

**Results:**

The plot illustrates how parameters simultaneously influenced the estimate. It can be combined with a Trellis plot in a so-called hybrid plot. Nested loop plots may also be applied to other criteria such as the variance of estimation.

**Conclusion:**

The nested loop plot, similar to a time series graph, summarizes all information about the results of a simulation study with respect to a chosen criterion in one picture and provides a suitable alternative or an addition to Trellis plots.

**Electronic supplementary material:**

The online version of this article (doi:10.1186/1471-2288-14-129) contains supplementary material, which is available to authorized users.

## Background

After a new statistical method has been developed, it is usually investigated in an extensive simulation study in order to evaluate its properties for future usage in real data applications. Particularly, it is important to compare the new statistical method to existing approaches. To this aim, a number of simulation parameters is varied, so that the value of the new method can be investigated in many different data scenarios. Moreover, there are usually several criteria. If the new method includes statistical hypothesis testing, size and power of the test are typically evaluated; if it is a method for parameter estimation, the criteria of interest are the absolute or relative bias, the mean squared error, the variance of estimation, and the coverage of the proposed confidence intervals [1–3].

For example, if each scenario is defined by setting a fixed combination of levels for five simulation parameters, each of which takes four levels, and we look at three criteria, we have for 4^5^=1024 different scenarios for each criterion. For presenting simulation results in a manuscript for publication, tables, figures or regression analyses can be used.

Information is often presented in a number of figures. A very suitable way of presentation is a Trellis plot. This is a rectangle of n × m plots (corresponding to two dimensions of the parameter space of the simulation study), each of which has a common third parameter as its horizontal axis and one of the criteria as vertical axis. Results from several methods can be compared in a single plot using different line types or colors for each.

Thus, three dimensions of the parameter space can be presented in one Trellis plot. If there are four, five or even more dimensions, as is often the case, lots of these plots must be presented. Alternatively, a selection is necessary due to restricted space. Trellis and related plots are frequent in the statistical literature, e.g., for testing and adjusting methods for publication bias [4–10] and meta-analysis in general [11–13].

It is also possible to present the results of a simulation study as a regression analysis, where the criteria are modelled in terms of the parameters. This way of presentation, however, is not frequent. One reason may be that the simulation parameters, originally often continuous variables, take only few levels in the simulation study. Thus they are treated as ordinal variables, which makes modelling and interpreting of the regression coefficients more challenging. Moreover, for adequate handling of variability within simulation scenarios, all simulation repetitions must be saved which is unusual. In addition, potential interactions must be modelled, so that many regression parameters are needed.

The method presented in this article originally arose from the desire to have all simulation results for a criterion in a single plot. The idea was to order all simulation scenarios in a lexicographical manner and arrange them consecutively on the horizontal axis of a plot. The criterion – e.g., treatment effect estimate, which shows potential bias, or variance of estimation, or mean squared error (MSE) – is shown on the vertical axis.

The paper is arranged as follows. In the next section, a motivating example is introduced. Based on this example, we explain the method in the subsequent section. In the ‘Results’ section the method is applied to the example. After discussing strengths and limitations in the ‘Discussion’ section, the paper ends in a Conclusion.

## Example: simulation study for selection bias in meta-analysis

**Figure 1:**
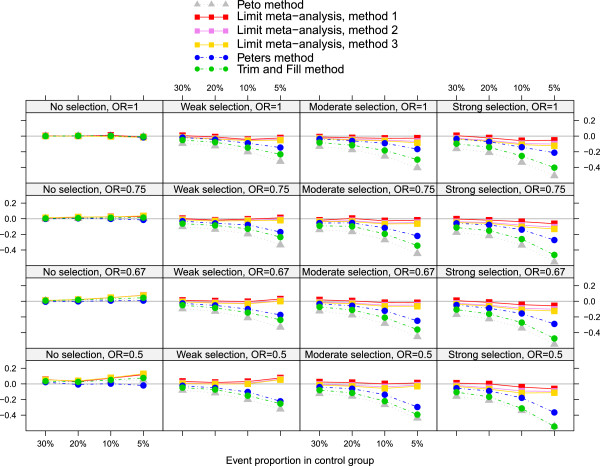
**Trellis plot.** Bias of treatment effect estimate on log odds ratio scale for the Peto method and five adjusting methods. Heterogeneity (*τ* ^2^=0.1) and number of studies (*k*=10) are fixed.

Before introducing the method, we present our motivating example. We performed a comprehensive simulation study on various treatment effect estimation methods for meta-analysis with binary outcome, where selection bias (e.g., publication bias, or other kinds of small-study effects) was suspected because of apparent funnel plot asymmetry [9, 10]. Figure 1 shows the bias of six treatment effect estimation methods for binary meta-analysis in several scenarios. Effect measure is the log odds ratio. One method is a standard approach of fixed effect meta-analysis, the Peto method [14]. The other five methods aim to estimate the treatment effect while adjusting for selection bias. We were interested in comparing three newly developed methods (here briefly called method 1, method 2 and method 3; for details see [9]) with two existing methods, the Peters method [8] and the Trim-and-Fill method [15].

We emphasize that for the purpose of this article, this particular simulation study only serves as an example. Background, methods and more details of the study are described elsewhere, where also similar Trellis plots are given, see ([9, 10], Figure two).

Figure 1 is a Trellis plot covering results of several simulation scenarios. The plot consists of 4 × 4 layers, differing by extent of selection (levels: no, weak, moderate, and strong selection; in horizontal direction) and true treatment effect (odds ratio: 1, 0.75, 0.67, 0.50; in vertical direction). Within each layer, the event proportion in the control group is varied (four levels 30%, 20%, 10% and 5%, on the horizontal axis in decreasing order). Criterion is the bias on the log odds scale, shown on the vertical axis. The six methods are presented as six lines marked by different colors. In summary, the Trellis plot contains information of 4 × 4 × 4 = 64 scenarios. In the underlying simulation study, however, two additional simulation parameters were varied: the heterogeneity variance parameter of the random effects model, *τ*^2^ (four levels: 0, 0.05, 0.10, 0.20) and the number of studies in meta-analysis (three levels: 5, 10, or 20 studies). In combination, this yields a total number of 4 × 4 × 4 × 4 × 3 = 768 scenarios. In the plot presented here, heterogeneity (*τ*^2^=0.1) and number of studies (*k*=10) are fixed. Twelve Trellis plots would have been needed to present all results.

In both earlier articles [9, 10], additional focus was on MSE and coverage of 95% confidence intervals. If all this information is presented, the number of figures triples, yielding 36 figures – far too many for most journal articles. Apart from this, it would be a enormous challenge for a reader to capture the full information contained in 36 figures. For these reasons, usually only a small part of simulation results is presented in a publication.

In this paper we focus on the novel plot, not the specialties of the motivating example. Nevertheless, the example is needed for explaining the features of the plot in detail. For this reason, we will refer to the example in each of the following sections.

## Methods

We start with a dataset containing as many rows as simulation scenarios considered. Let there be *P* simulation parameters *p*=1,…,*P*, and let parameter *p* have levels *x*_*pj*_(*j*=1,*n*_*p*_) where *n*_*p*_ is the number of levels of parameter *p*. Then the total number of scenarios *N* (and thus the number of rows in the dataset) is given by . Each scenario is characterized by a unique combination of *P* values .

Hence, each parameter adds one column to the data set. For *M* methods and *C* criteria, simulation results will add *M*×*C* columns. Accordingly, the dataset with simulation results has a structure as shown in Table 1.

**Table 1:** **Structure of dataset with simulation results**

Scenario	Simulation	First	Second	*⋯*
	parameters	criterion	criterion	
	*1 ⋯ P*	*C*_*11*_ *⋯ C*_*1M*_	*C*_*21*_ *⋯ C*_*2M*_	
1	*x*_11_ ⋯ *x*_*P*1_	*c*_111_ ⋯ *c*_1*M*1_	*c*_211_ ⋯ *c*_2*M*1_	⋯
⋮	⋮	⋮	⋮	⋮
N	*x*_1*N*_ ⋯ *x*_*PN*_	*c*_11*N*_ ⋯ *c*_1*MN*_	*c*_21*N*_ ⋯ *c*_2*MN*_	⋯

In our example, the first criterion is the average treatment effect estimate from all repetitions within the same simulation scenario which is available for six statistical methods. Additional criteria are the observed variance or the standard error of this estimate, the MSE and the coverage of 95% confidence intervals. For statistical tests, the observed rejection frequency under the given scenario is typically recorded. This is interpreted as the type I error if the scenario belongs to the null hypothesis, and as the power if the scenario belongs to the alternative hypothesis. For other statistical methods, other types of measures may be appropriate. The dataset with simulation results for our example is attached as Additional file 1.

The underlying idea of the new ‘nested loop plot’ is to reorder simulation scenarios into a lexicographical order and arrange them consecutively on the horizontal axis of a plot, whereas the criterion to evaluate simulation results – e.g., treatment effect estimate, which shows potential bias, or variance of estimation, or mean squared error (MSE) – is presented on the vertical axis.

Lexicographical order means the following. First, for the *P* simulation parameters we choose one of *P*! possible orders. The chosen order defines a nested sequence of loops. Secondly, within each loop, corresponding to a simulation parameter *p*, we define how to sort its *n*_*p*_ levels. Whereas for the ranking of the levels there is often a natural choice (e.g., the number of studies in a meta-analysis is ordered from large to small), the order of the parameters is in principle arbitrary. Therefore it should be well considered. We recommend to sort the simulation dataset in such a way that the simulation parameter with the largest influence on the criterion of interest is considered first, and so forth.

### Example

In our example, focus was on estimation of the treatment effect given as log odds ratio. Accordingly, the true odds ratio was chosen to serve as the first parameter in the order. We started with ‘no treatment effect’, that is an odds ratio of one, and increased the treatment effect by decreasing the odds ratio. Without loss of generality, only odds ratios less or equal to one were considered. As the second simulation parameter, we chose the selection parameter in increasing order, as the main issue in our study was selection. The third to fifth nested loops were chosen to be the event proportion in the control group (decreasing), the heterogeneity variance (increasing), and the number of studies in the meta-analysis (decreasing). The leading principle here was that we wanted to start with scenarios where the estimation was likely to be more accurate and end up with scenarios probably more difficult to predict.

R functions for reordering the simulation dataset as well as generating the nested loop plot are given in Additional file 2, together with an example of its use in Additional file 3.

## Results

**Figure 2:**
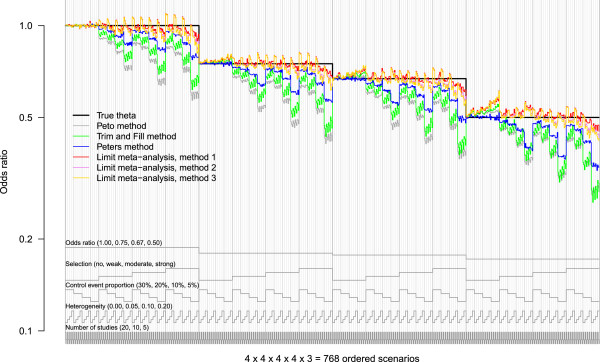
**Nested loop plot of treatment effect estimates.** 768 simulation scenarios in total, order from outer to inner loops: Treatment effect (4 levels, increasing); selection (4 levels, increasing); control event proportion (4 levels, decreasing); heterogeneity (4 levels, increasing); number of studies (3 levels, decreasing). The periodic turn of the loops is illustrated by the gray lines at the bottom of the plot.

The method and its application are best explained using the motivating example. The nested loop plot for the example considering all 768 simulation scenarios is shown in Figure 2. The simulation results were arranged according to the order of loops described in the last section.

We here focus on the accuracy of the treatment effect estimate as our criterion of interest, depending on the estimating method and the simulation scenario. The true treatment effect for each scenario was known and served as a benchmark (black horizontal line). The difference of each estimate to this benchmark is the observed bias. We summarize the information in the plot as follows.

### Odds ratios

Differences in accuracy between the various odds ratios were present, but not marked; see below.

### No selection

With respect to selection, we observe marked differences. If there was no selection (the left quarter in each section), the Peto method and the Peters method worked well. The other methods yielded estimates that were slightly biased upwards, the more so, the more the odds ratio deviated from one, and the less the control event proportion was. These observations confirm earlier results from the literature: estimation becomes more biased if events are rare, and the Peto method is recommendable in this setting, except if the odds ratio differs markedly from one [11, 16]. The adjusting methods 1, 2 and 3 were even more biased upwards.

### Increasing selection

If selection increased, the Peto method became markedly biased. Also the Trim and Fill method and the Peters method showed large bias, particularly if the event was very rare (5%). The methods 1, 2 and 3 were also biased, but not so much and often in the opposite direction, except for strong selection.

### Event proportion

As said before, all methods showed large bias if events were rare, particularly if accompanied by other problems, such as strong selection and also a large treatment effect.

### Heterogeneity

The influence of heterogeneity was less marked than that of other simulation parameters. For the Peters method, heterogeneity had the least influence. For the Peto method and the Trim and Fill method, increasing heterogeneity led to less bias. For the methods 1, 2 and 3, increasing heterogeneity led to treatment effect estimates farther from one, which increased bias if selection was strong.

### Number of studies

A closer look at the smallest unit of the plot showed that, other parameters held fixed, the bias tended to slightly increase if the number of studies in the meta-analysis decreased.

### Interpretation

Whereas the first four of these conclusions could have just as well been drawn from Figure 1, at least for the fixed combination of heterogeneity (*τ*^2^=0.10) and number of studies (*k*=10), judgment of the latter two points was not possible from Figure 1 alone, as other levels of heterogeneity and number of studies were not considered.

### Hybrid plot

**Figure 3:**
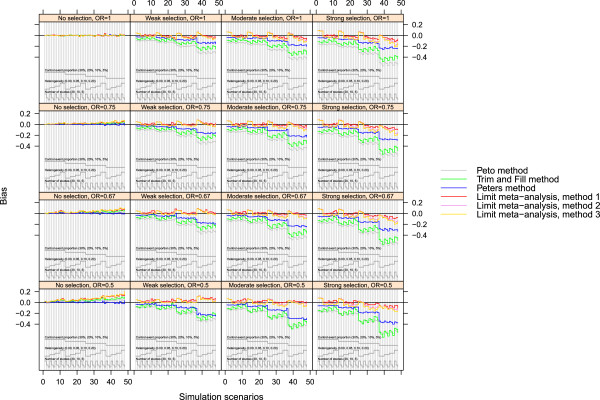
**Hybrid nested loop plot.** A combination of trellis plot and nested loop plot showing 4×4×3=48 scenarios per layer: event proportion (5%, 10%, 20%, 30%); heterogeneity (*τ* ^2^=0,0.05,0.1,0.2); number of studies (5, 10, 20).

As a variant of a nested loop plot, we also produced a sort of hybrid between Trellis and nested loop plot which is shown in Figure 3. For this figure, the 4×4 panels of the Trellis plot (Figure 1) and their order were kept as before, but in each panel all 4×4×3=48 scenarios are presented in the same order as in Figure 2). R code for producing a hybrid plot is found in Additional files 2 and 3 in the web appendix.

## Discussion

We proposed a novel figure type – the nested loop plot – to illustrate the results of simulation studies with many simulation parameters. The nested loop plot can be used as an alternative or in addition to established plots like Trellis plots. It is particularly useful if there are more than three simulation parameters, which makes it necessary to show more than one Trellis plot in order to present all results graphically. The basic idea of the nested loop plot is comparable to that of a time series diagram showing, for example, meteorological data, or secular and seasonal variations of the incidence of an infectious disease, or of the number of patients seeking medical advice. The outer loops (e.g., decades and years) correspond to secular trends, such as a longtime increase or decrease of the incidence, whereas the inner loops (months, weeks, days or even hours) show seasonal, weekly or hourly variations of the criterion of interest.

The diagram is especially suited to illustrate the accuracy and bias of parameter estimation. In this case, the vertical axis is given by the scale of the parameter to be estimated. This parameter should correspond to the first-level parameter in the order, and the true value of this parameter is clearly marked in the diagram as a step function, such as the true odds ratio in Figure 2. In this case, the nested loop plot may replace dozens of Trellis plots, particularly if the fourth, fifth and so forth simulation parameter have many levels.

### Interactions

An advantage of our approach as compared to regression analyses is that it is not necessary to model interactions between parameters explicitly. If there are interactions, they may be readily seen as patterns in the nested loop plot. For example, in Figure 2 it is seen that for odds ratios different from one and no selection, the Peto method and the Trim and Fill method tend to be upwardly biased (the more so, the lower the control event proportion is), whereas they are downwardly biased, if there is selection (the more so, the lower the control event proportion is). In other words, we observe an interaction between the extent of selection and the control event proportion. This could be confirmed by a regression model.

We produced nested loop plots for another simulation study comparing tests for funnel plot asymmetry [7]. The null hypothesis was ‘no selection’, and the criterion was the rejection probability of the tests. This represents the type I error, if the null hypothesis was true (i.e., if there was in fact no selection), and the power in case of the alternative hypothesis being true. The nested loop plot gave a clear picture of the results (not shown). However, it offered no great advantage over three or four Trellis plots, as there were only four parameters varied in this simulation study: selection (4 levels); number of studies (3 levels); heterogeneity (3 levels); true treatment effect (3 levels).

### Ordering the loops

The order of the loops is arbitrary. However, this likewise holds for Trellis plots, as the choice of axes and layers is subject to subjective decisions, too. It is proposed to choose the true parameter as the first level of variation (outer loop). For the other levels, the principle is to sort parameters by the magnitude of their influence on the criterion of interest, with parameters with greater influence coming first. The influence can be investigated by a regression model. The aim is to avoid a large number of extremely close peaks in order to have curves as smooth as possible. R functions provided in the appendix can be used to easily change the order of simulation parameters.

### Ordering the parameter levels within loops

The order of the levels within the loops is also arbitrary. The principle we used here is ‘from simple to difficult’. For example, in the framework of treatment effect estimation in meta-analyses with binary outcome it is often more difficult to estimate the treatment effect if there are only few studies, if events are rare, or if heterogeneity is large. Therefore, we ordered the levels by decreasing the number of studies, decreasing the control event proportion and increasing the heterogeneity.

It is possible to sort loops and parameters within loops on the basis of the results of a regression model. The order may then be determined using the magnitude and the sign of the regression coefficients. In principle, this might even be done automatically. However, since the magnitude of the regression coefficients depends on the scaling of the various parameters, the coefficients are not directly comparable.

Again, R functions provided in the appendix can be used to easily reorder the levels of simulation parameters.

### Limitations

The nested loop plot has a number of limitations.

#### Number of parameters and scenarios

Of course, the number of simulation parameters, levels of parameters and the total number of scenarios is not unlimited. In our example, we considered five parameters, leading to 768 scenarios. We think that no more than six parameters and about 1000 scenarios can be presented in one plot, additionally depending on the extent of variation, particularly the lack of monotonicity between adjacent scenarios. The nested loop plot is the less readable, the more peaks are observed. By contrast, the hybrid plot offers the chance to present more than 1000 scenarios.

#### Other criteria

**Figure 4:**
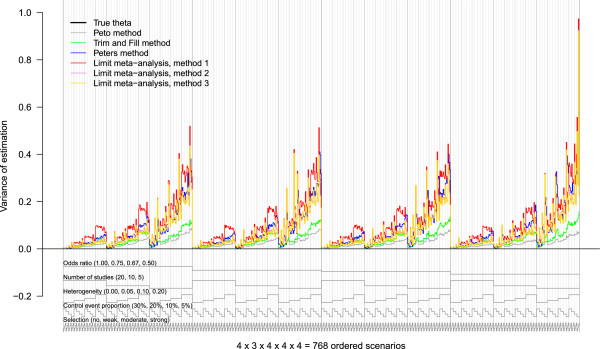
**Nested loop plot of the variance of estimation.** 768 simulation scenarios in total, order from outer to inner loops: Treatment effect (4 levels, increasing); number of studies (3 levels, decreasing); heterogeneity (4 levels, increasing); control event proportion (4 levels, decreasing); selection (4 levels, increasing). The periodic turn of the loops is illustrated by the gray lines at the bottom of the plot.

In our experience, the nested loop plot is somewhat less suitable for criteria such as variance of estimation, MSE, or coverage of confidence intervals. At least in our simulation study, the reason was that all parameters had a large influence on these criteria, as shown by Figure 4 for the variance of estimation. We obtained a large number of close peaks, regardless which order we had chosen, which rendered the plot confusing.

#### Monte Carlo error

Of note, there are important questions that are not answered by looking at a nested loop plot. One of these is the error associated with the estimated quantities, the so-called Monte Carlo error [2]. However, this is a disadvantage the proposed plot has in common with the Trellis plot. It is recommended to present this information in addition to a nested loop plot.

## Conclusion

The nested loop plot, similar to a time series graph, summarizes all results of a simulation study with respect to a chosen criterion in one picture. It provides a suitable alternative to Trellis plots, possibly in combination with them (hybrid nested loop plot), at least for a first but comprehensive overview.

## Electronic supplementary material

Additional file 1: **Data file of example data (** res.rda **).** This file (res.rda) contains the data of the example. (ZIP 238 KB)

Additional file 2: **R functions.** The filename is nestedloop.R, containing three R functions. R function nestedloop can be used to reorder a dataset with simulation results. Argument x is the simulation dataset, argument varnames is a character string giving the variable names of simulation parameters used for ordering. Both arguments x and varnames are mandatory. Arguments sign and varlabels are optional. R function lines.nestedloop can be used to plot vertical and reference lines in an nested loop plot. Only argument x is mandatory. R function panel.nestedloop can be used to plot vertical and reference lines in a hybrid plot. Only argument x is mandatory. (ZIP 1 KB)

Additional file 3: **R commands for the example.** This file (example-fig2-fig3-new.R) contains the R commands for the example (nested loop plot, Figure 2; hybrid plot, Figure 3). (ZIP 2 KB)
